# A molecular study of modern oil paintings: investigating the role of dicarboxylic acids in the water sensitivity of modern oil paints†

**DOI:** 10.1039/c7ra13364b

**Published:** 2018-02-06

**Authors:** Donatella Banti, Jacopo La Nasa, Anna Lluveras Tenorio, Francesca Modugno, Klaas Jan van den Berg, Judith Lee, Bronwyn Ormsby, Aviva Burnstock, Ilaria Bonaduce

**Affiliations:** Courtauld Institute of Art Somerset House, Strand London WC2R 0RN UK; Department of Chemistry and Industrial Chemistry, University of Pisa Via Moruzzi 13-56124 Pisa Italy ilaria.bonaduce@unipi.it; Cultural Heritage Agency of the Netherlands (RCE) Hobbemastraat 22 1071 ZC Amsterdam Netherlands; Conservation Department, Tate, Millbank London SW1P 4RG UK

## Abstract

The 20th century has seen a significant evolution in artists' paint formulation and technology which is likely to relate to the new conservation challenges frequently presented by modern oil paintings, including unpredictable water- and solvent-sensitivity. This study examined the molecular causes and mechanisms behind these types of modern oil paint vulnerability. Research performed up to now has suggested a correlation between the occurrence of water sensitivity and the presence of relatively high amounts of extractable free dicarboxylic acids. To explore this further, as well as the influence of paint formulation, a set of model paint samples, produced in 2006 using commercial tube paints to which known amounts of additives were added, were analysed. The samples were tested for water sensitivity by aqueous swabbing and characterised using transmission Fourier Transform-Infra Red spectroscopy (FTIR) to determine the molecular composition of the main paint constituents, High Performance Liquid Chromatography-Mass Spectrometry (HPLC-MS), to identify the type(s) of drying oils used as binders, and Gas Chromatography-Mass Spectrometry (GC-MS) using a recently developed analytical procedure that can discriminate and quantify free fatty and dicarboxylic acids, as well as their corresponding metal soaps (carboxylates of fatty and dicarboxylic acids). The results indicated that the addition of small amounts of additives can influence the water sensitivity of an oil paint, as well as its molecular composition. Additionally the nature of the ionomeric/polymeric network appears to be a significant determining factor in the development of water sensitivity.

## Abstract 2

Quantitative mass spectrometry was used to investigate the role played by dicarboxylic acids in the water sensitivity of modern oil paints.

## Graphical abstract

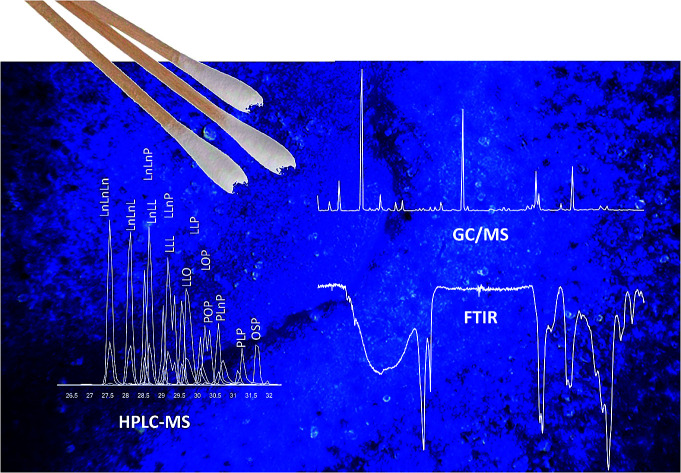

## 1. Introduction

From the 18th century, artists' oil paint production evolved from small-scale production in studios, where paints were prepared from raw materials according to traditional and often well-protected recipes, to Colourmen, where paints were prepared in larger batches according to more standardised procedures. In the nineteenth century, scientific and technological advancements enabled the mass production of artist's oil paint. The introduction of the collapsible paint tube in 1841, necessitated an increase in the complexity of paint formulation in order to satisfy rheology and durability criteria for oil paints requiring a longer shelf life.^1^ From the middle of the twentieth century, new synthetic polymers such as acrylic and alkyd media became available, although 20^th^ century artists have continued to use paints based on drying oils:^2^ indeed, a recent survey at Tate found that more than 70% of their modern paintings contain oil media.^3^ Modern artists' paints, including oil paints, can incorporate additions of metal salts, metal soaps, a variety of dispersion agents, plasticizers, fillers and surfactants, amongst other materials, to influence specific properties such as rheology, stability, drying rate, and colour.^1,4–8^ Among these, metal soaps and/or free fatty acids (FFA) were added to oil-based paints as dispersing agents in order to improve the wetting properties of the pigments, and their ability to be homogeneously dispersed in the oil binder.^9–12^

Some modern oil paintings are now beginning to present challenges for conservation:^13^ deterioration due to material composition and exposure to environment have led to surface changes, and in some cases, the migration and aggregation of chemical species occurs, leading to a range of issues such as the formation of vulnerable ‘medium skins’ on paint surfaces, efflorescence, protrusions, colour change and paint delamination, as well as water- and solvent-sensitivity of paint surfaces.

The sensitivity of unvarnished modern oil paintings to standard surface cleaning methods such as swabbing with water or saliva has been reported by conservators working both in museums and private collections.^9^ In the last ten years this problem has been the focus of interdisciplinary research involving conservators and scientists.^3,9,13,14^ Research consortia such as the Modern Oil Research Consortium (MORC)‡‡Tate, Modern Oil Research Consortium, Available at: http://www.tate.org.uk/about/projects/modern-oils-researchconsortium and the European Joint Program Initiative (JPI) Heritage Plus funded project on the Cleaning of Modern Oil Paints (CMOP), reflect the attention that this issue is currently receiving.^14^

Water sensitivity has now been identified in model oil paint samples prepared from raw materials^13,15,16^{Tempest, 2010 #16}, in samples taken from batches of manufactured paint,^14^ and in numerous paintings.^16–18^ Sensitivity may be limited to certain colours and passages, may affect the whole surface of a painting, may be specific to some paint brands or lines, and may affect specific pigments across several brands.^15,18^

One key area of investigation involves the exploration of how paint formulations and additives may influence paint ageing and degradation processes.^3^ The proven causes of sensitivity thus far identified include the formation of magnesium sulphate heptahydrate (epsomite) on some paint surfaces, as a result of the presence of magnesium carbonate in paint formulations, which can react with sulphur dioxide from the atmosphere.^13–15,17^ The presence of under-bound or lean paints can also contribute to sensitivity, which is may derive from artists' technique.^3,9^ However, water sensitivity has also been observed in many well-bound (or ‘fat’) paints.

A related study identified a strong relationship between water sensitivity and pigment type: paints formulated with zinc oxide and/or lead were consistently non-water sensitive.^14,19^ The study also demonstrated that in general, water sensitive paints were not associated with a higher degree of oxidation compared with non-sensitive paints, although some highly oxidised paints (mostly containing Fe and Mn pigments) were often noted as water sensitive.^14^ In general, irrespective of the overall degree of oxidation of the paint, water extracted a relatively higher amount of free dicarboxylic acids from water-paints than from non-water sensitive paints.^14^ Dicarboxylic acids, which are the products of oxidation of a drying oil,^20^ exhibit a certain degree of water solubility: 2400 mg L^−1^ (at 20 °C) for azelaic acid, *versus* 0.04 mg L^−1^ (at 25 °C) for palmitic acid. This lead to the hypothesis that a sample rich in relatively high amounts of free – unbound and non-saponified – dicarboxylic acids, may be water sensitive during cleaning, *via* swelling and weakening the paint structure. On the other hand, where dicarboxylic acids are present as metal soaps (as opposed to in free form), they are more likely to form a relatively stable, three-dimensional metal coordinated network, due to their chain building ability.^21^

In this study, these hypotheses were investigated further by exploring the relationship between water sensitivity and the composition and proportions of free and saponified dicarboxylic acids. A set of model cobalt blue and raw sienna paint samples exhibiting varying degrees of water sensitivity were characterised using transmission infrared spectroscopy (FTIR), liquid chromatography mass spectrometry (LC-MS) and gas chromatography mass spectrometry (GC-MS), the latter using a recently developed analytical procedure that can discriminate and quantitate free fatty and dicarboxylic acids, and their corresponding metal soaps.^22^

## 2. Materials and methods

### 2.1. Samples

Model paint samples belong to a collection prepared in 2006 at the Netherlands Institute for Cultural Heritage (ICN) in 2006 using Winsor & Newton Artists' Oil Colours (WN) and Talens Rembrandt Oil Colours (TA), with cobalt blue (CB) and raw sienna (RS) as pigments. Aliquots of paint were applied unadulterated or with addition of one additive on Melinex® supports according to a standardised procedure, the details of which have been reported elsewhere.^11,18^ The model paint samples were light aged under indoor conditions at high lux§§Artificial ageing was done at Stichting Restauratie Atelier Limburg (SRAL). Illumination was provided by 36 W Philips colour 96.5 fluorescent lamps, with UV filtering (transmission 15 watt per lumen), rendering a measured output of 10 000 lux at the sample surface. Temperature was 25 °C and RH 60% on average and the total ageing time of 1390 hours. The total ageing time of 1390 hours is calculated to be equivalent to twenty-four years of exhibition in recommended museum conditions, 200 lux, eight hours a day, assuming reciprocity.^11,20^, and subsequently stored indoor in room ambient conditions until 2012, when they were dismounted and kept in darkness inside drawers.^23^

The model paint samples used in this study are listed in Table 1 and additives used are 2% aluminium stearate (AS), 2% zinc stearate (ZS), 2% free fatty acid (FA)–heptadecanoic acid (margaric acid). It is noted that the samples selected for this study did not contain detectable amounts of epsomite.^23^ At low magnification, the surface of samples appeared to be well bound.^23^ As a result, water sensitivity of these samples could not be ascribed to the presence of soluble salts or under-bound paints.

**Table 1:** Composition of model paint samples as obtained from the manufacturer and elemental analysis

Manufacturer/series	Brand colour (and number)/pigment used¶	Pigment chemical composition or formula	Elemental composition of unadulterated model paint samples^a^	Additives added during preparation of model paint samples in 2006 (ref. 18)	Model paint acronyms
Winsor & Newton/Artists' Oil Colour	Cobalt blue deep (180)/PB74	Co–Zn silicate (Co, Zn)_2_SiO_4_	Co, Zn, si, O, Mg, C, Ba	None	WNCB
				2% margaric acid	WNCBFA
				2% Zn stearate	WNCBZS
				2% Al stearate	WNCBAS
Talens/Rembrandt Oil Colour	Cobalt blue (513)/PB28	Cobalt aluminate (blue Spinel) (CoAl_2_O_4_)	Co, Al, O, Ca, C, Zn, Mg	None	TACB
				2% margaric acid	TACBFA
				2% Zn stearate	TACBZS
				2% Al stearate	TACBAS
Winsor & Newton/Artists' Oil Colour	Raw sienna (552)/PY42, PY43	Natural iron oxide, (PY43: Fe_2_O_3_·H_2_O with impurities) synthetic iron oxide (PY42: Fe_2_O_3_·H_2_O)	Fe, O, Ca, C, K, Al, Si (Zn)	None	WNRS
				2% margaric acid	WNRSFA
				2% Zn stearate	WNRSZS
				2% Al stearate	WNRSAS

a Results obtained *via* SEM-EDX analysis of the unadulterated model paint samples.^23^

It is noted that the pigments used in the cobalt clue paints of Winsor and Newton and Talens are different. Pigment PB74 Co–Zn silicate is used in the Winsor and Newton cobalt blue deep paint, while pigment PB28, cobalt aluminate is used in the Talens cobalt blue paint.

### 2.2. Water sensitivity tests

The test used to establish the sensitivity of the paint surfaces to water was based on a semi-standardised method, used in previous studies, involving the rolled application of dampened cotton wool swabs to the paint surface.^18,24^ The swab roll tests were performed twice, and evaluation of sensitivity was based on the average number of swabs rolls that could be applied to the paint surface until pigment particles were picked up onto the swab. Sensitivity criteria used are reported in Table 2.

**Table 2:** Sensitivity criteria used to determine sensitivity of paint to swab rolling using deionised water

Water sensitivity criteria	Nr swab rolls necessary to remove the paint	Numerical indicator
Not sensitive	≥31	1
Moderately sensitive	21–30	2
Sensitive	11–20	3
Very sensitive	≤10	4

The paints used in this study were selected from a larger batch based on differences in water sensitivity behaviours. The selected paints range from very¶For Winsor and Newton information were taken directly from the tubes. For Talens the information was available at: https://www.royaltalens.com/media/1412025/Consumentenfolder_olieverf_EN.pdf [accessed 15/12/2016]. Full formula and other information on the pigments can be found in the Color of Art Pigment Database; http://www.artiscreation.com/[accessed 15/12/2016]. sensitive to non-sensitive, and include samples whose water sensitivity characteristics had changed since earlier tests were carried out.^11,23^.

### 2.3. GC-MS

Fragments of the model paint samples (600–800 μg) were subjected to a double derivatisation procedure in order to separately analyse and quantify both free fatty acids and carboxylates of fatty and dicarboxylic acid (not bound to the polymeric network, nor to glycerides).^22^||||J. La Nasa, A. Lluveras-Tenorio, F. Modugno, I. Bonaduce, “Two-steps analytical approach for the characterization and quantification of metal soaps and resinates in paint samples”, in preparation. Analyses were performed in triplicates for each model paint, after crushing paint fragments in an agate mortar to ensure sample homogeneity. Samples were augmented with a tridecanoic acid solution (5 μL, 125.8 ppm in isooctane) as an internal standard and then subjected to the first derivatization for the GC-MS analysis by adding derivatising agent hexamethyldisilazane (HMDS, 20 μL, Sigma-Aldrich) and *iso*-octane solvent (50 μL, Sigma-Aldrich), and heated to 60 °C for 30 min. A second internal standard, hexadecane, (97.23 ppm in isooctane) was also added before injection. An aliquot (2 μL) of the supernatant solution was injected into the GC-MS. The residual solution was then dried under a nitrogen flow, and subsequently derivatised with the second derivatising agent, bis trimethylsilyltrifluoroacetamide (BSTFA, 20 μL, Sigma-Aldrich) in *iso*-octane (50 μL), heated at 78 °C for 80 minutes. An aliquot (2 μL) of this solution was then injected into the GC-MS. Quantitation of lauric, suberic, azelaic, myristic, sebacic, palmitic, oleic and stearic acids, was performed using calibration curves built using standard solutions containing a mixture of the analytes in acetone (Sigma-Aldrich) in the range of 1–100 μg g^−1^.

Analyses were performed with a GC-MS instrumentation consisting of an Agilent Technologies 6890N gas Chromatograph coupled with a 5973 mass selective detector single-quadrupole mass spectrometer. Samples were injected in splitless mode at 280 °C and gas chromatography (GC) separation was performed on a fused silica capillary column HP-5MS (J&W Scientific, Agilent Technologies, stationary phase 5% diphenyl 95% dimethyl-polysiloxane, 30 m length, 0.25 mm i.d., 0.25 μm film thickness). Chromatographic conditions were: initial temperature 80 °C, 2 min isothermal, 20 °C min^−1^ up to 280 °C, 10 min isothermal. MS parameters: electron impact ionization (EI, 70 eV) in positive mode; ion source temperature 230 °C; scan range 50–700 *m*/*z*; interface temperature 280 °C. Analyses were performed both in Selected Ion Monitoring (SIM) and Total Ion Current (TIC) modes.

### 2.4. FTIR

The bulk of the model paint samples were analysed using transmission FTIR spectroscopic analysis, using a Thermo scientific Nicolet iN10 MX microscope with a single diamond cell, equipped with an MCT-A/CdTe detector. 64 scans were collected at a resolution of 4 cm^−1^ across a wavenumber range of 4000 to 675 cm^−1^. Paint fragments were applied to a single diamond cell and rolled flat using a steel roller. Data were processed using Omnic 8 software.

## 3. Results and discussion

### 3.1. Water sensitivity tests

The unadulterated WNCB paint was very sensitive to water, but the addition of 2% margaric acid or 2% aluminium stearate or 2% zinc stearate to these paints generally caused a slight decrease in sensitivity. The TACB (cobalt blue) paints were found to be only moderately sensitive to water. The effect of the addition of Al and Zn stearates on the Talens cobalt blue paint resulted in a decrease of the sensitivity to water swabbing. However, the addition of 2% margaric acid increased the sensitivity to water when compared to the same paint without additives. All of the WNRS (raw sienna) series were not sensitive to swabbing with deionised water, and in this case, the presence of the additives did not result in significant changes in water sensitivity. The results of the water sensitivity tests are summarised in Table 3.

**Table 3:** Results of water sensitivity tests to water

Sample	Sensitivity to water
WNCB	4
WNCBFA	3
WNCBAS	3
WNCBZS	3
TACB	2
TACBFA	3
TACBAS	1
TACBZS	1
WNRS	1
WNRSFA	1
WNRSAS	1
WNRSZS	1

### 3.2. FTIR

**Fig. 1:**
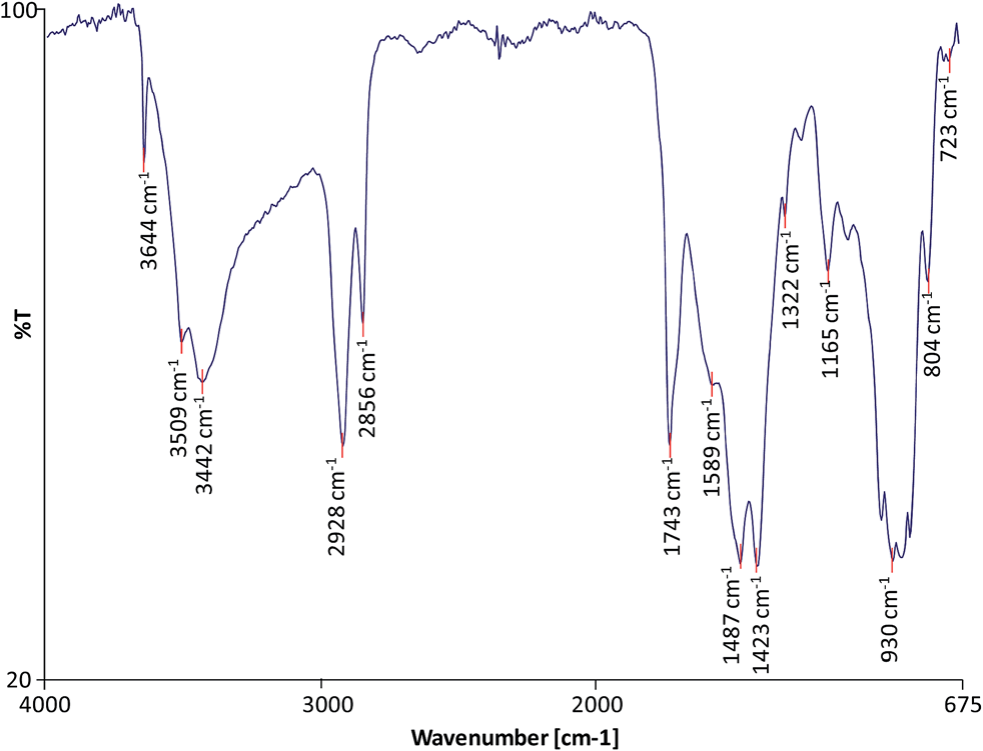
FTIR spectrum of sample WNCB.

**Fig. 2:**
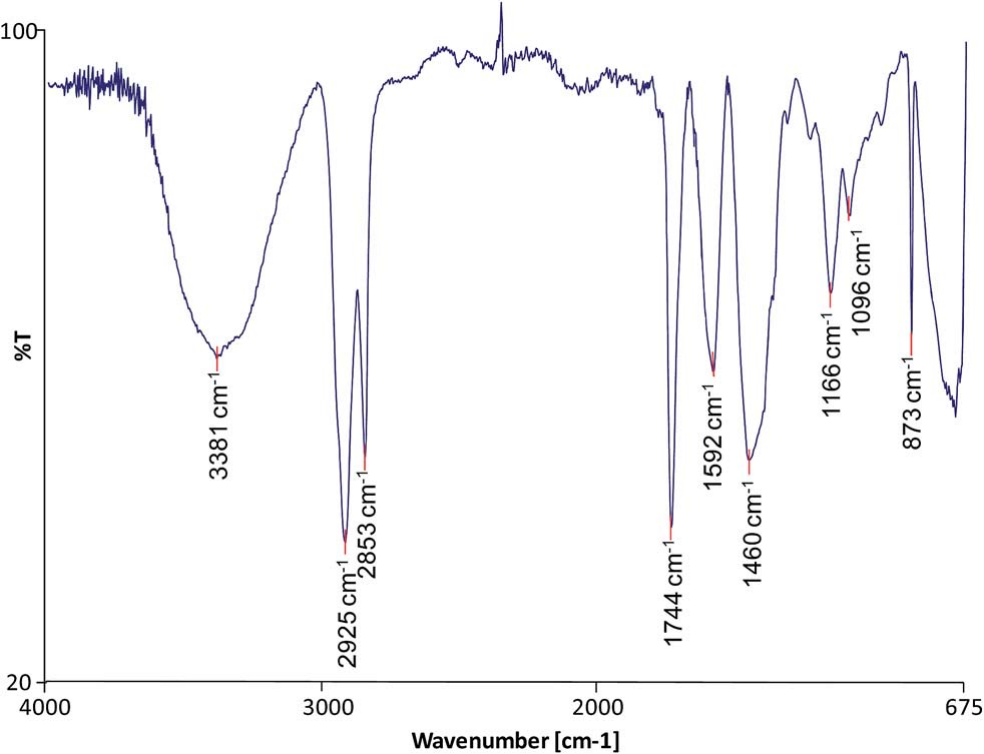
FTIR spectrum of sample TACB.

**Fig. 3:**
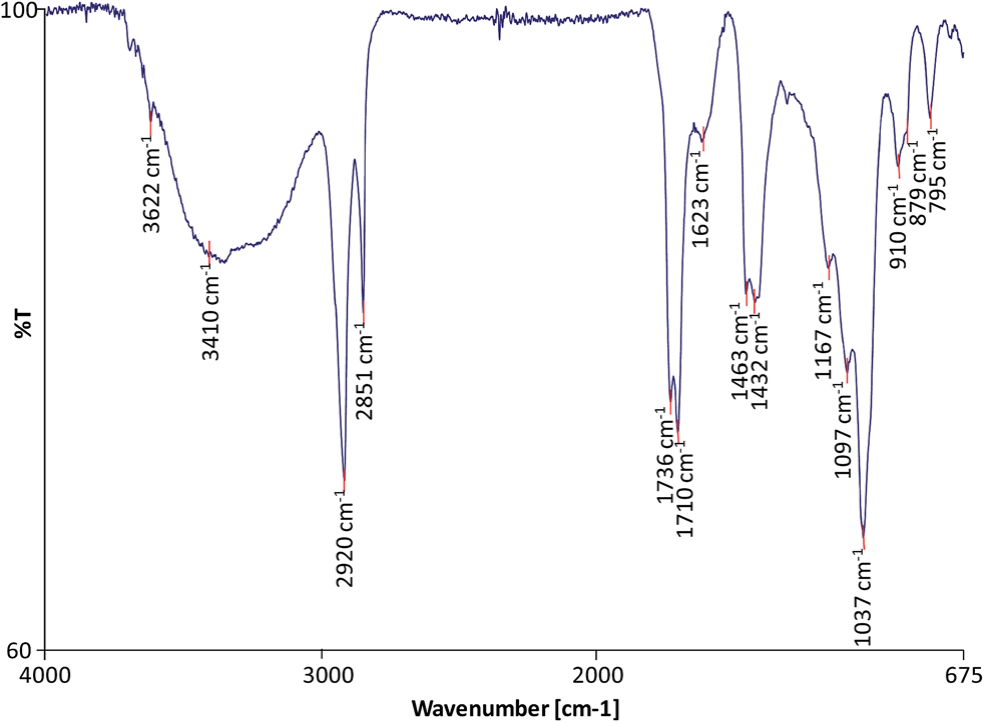
FTIR spectrum of sample WNRS.

FTIR was used to characterise the molecular composition of the selected model paint samples and to gain information on the degree of hydrolysis of the paint and formation of metal carboxylates. The spectra of the unadulterated samples are presented in Fig. 1–3.

All the spectra show the characteristic bands of an oil binder: the broad band centered at *ca.* 3440 cm^−1^ is assigned to the stretching of alcohol and hydroperoxide bonds, the band at *ca.* 1740 cm^−1^ is assigned to the ester stretching, and the bands corresponding to the CH stretching are *ca.* 2928 and *ca.* 2860 cm^−1^. The FTIR spectrum of sample WNRS (Fig. 3) shows also the presence of a band at 1710 cm^−1^ corresponding to the C=O stretching vibration related to the formation of free fatty acids as a result of the hydrolysis of triglycerides, and, to a certain extent, oxidation with formation of dicarboxylic acids. This is in agreement with the results obtained for other paint reconstructions consisting of linseed oil and Fe based pigments (red ochre, Prussian blue and red bole)^25^ confirming the observation that Fe-based pigments promote the hydrolysis of triglycerides.^26^ Free fatty acids could not be distinguished in WNCB and TACB (Fig. 1 and 2), however this has been observed in other paints containing Co and Zn containing-pigments (zinc white and cobalt green).^14,25,27^ A split carbonyl band with absorptions at *ca.*1740 cm^−1^ and *ca.* 1710 cm^−1^ has been associated with water sensitive oil paints in a related study.^14,27^

The spectra of both WNCB and TACB (Fig. 1 and 2) showed the presence of a broad band centred at *ca.* 1590 cm^−1^. Amorphous Zn and Pb soaps are characterised by a broad band at *ca.* 1590 cm^−1^ (ref. 28) and *ca.* 1580 cm^−1^ (ref. 29) respectively, which are absorptions shifted ∼45 cm^−1^ toward higher wavenumbers with respect to the absorptions of their corresponding crystalline Zn and Pb soaps at *ca.* 1540 cm^−1^.^30,31^ Assuming that the amorphous metal soap band for Co stearates is associated with a similar shift in wavenumber with respect to its crystalline form, expected at *ca.* 1540 cm^−1^,^32^ then the broad band at *ca.* 1590 cm^−1^ (Fig. 1 and 2) may tentatively be ascribed to amorphous carboxylates of Zn and/or Co. The presence of the sharp band in the spectrum of WNCB (Fig. 1) at *ca.* 3650 cm^−1^, together with the bands at *ca.* 3509 and *ca.* 3442, *ca.* 1487 and *ca.* 1423 cm^−1^ related to the CO_3_^2−^ vibration, and the sharp band at *ca.* 803 cm^−1^, correspond to hydromagnesite (a form of magnesium carbonate) known to be used in Winsor and Newton oil paints.^33^ The bands at *ca.* 1430 and *ca.* 873 cm^−1^ in the TACB spectrum are related to the presence of calcium carbonate (CaCO_3_). This is in agreement with the presence of Mg and Ca in the elemental composition of these paint layers (Table 1). The use of the Co–Zn silicate–silicate pigment (PB74) in WNCB, (Table 1), is further confirmed by the broad band centered at *ca.* 930 cm^−1^ and that at *ca.* 723 cm^−1^ (Fig. 1).

Sample WNRS shows the characteristic bands of raw sienna: *ca.* 3687, *ca.* 3621, *ca.* 1623, *ca.* 1037, *ca.* 910, 887 and 795 cm^−1^; related to the presence of kaolinite in the naturally sourced pigment (Fig. 3). Despite the presence of Ba in the WNCB, and Zn and Mg in TACB and traces of Zn in WNRS (Table 1),^23^ their molecular composition could not be ascertained from the FTIR spectra. This is due to the fact that their diagnostic bands might be masked by other more abundant bands, and/or these compounds might be present in amounts below the detection limit, or beyond the acquisition wave range (oxides, sulphides, *etc.*). Ba is likely to originate from barium sulphate – a common paint extender, Mg to magnesium carbonate and Zn may originate from added stearates or ZnO, commonly added to paint formulations. Added Zn stearates would be in their crystalline form and would thus show a sharp band at *ca.* 1536 cm^−1^,^25^ which is not visible in the spectrum of sample TACB. The sharp band at *ca.* 1321 cm^−1^ in the spectrum of sample WNCB (Fig. 1) may be due to the presence of oxalates or a C–O absorption from the oil medium. No other bands were present that would help to confirm the identification of the oxalate type. Mg, Zn and Co oxalates have sharp bands in the range 1320–1325 cm^−1^, which were not detected in these samples.^34,35^

### 3.3. GC-MS

High-Performance Liquid Chromatography coupled to Electrospray Ionisation and Quadrupole Time-of-Flight Mass Spectrometry (HPLC-ESI-Q-ToF) was used for triglyceride profiling^36–41^ to identify the drying oil(s) used as paint binders, the results of which are discussed in the electronic ESI.† In summary, all of the samples contained a mixture of drying and semi-drying oils: TACB contained safflower oil, WNCB a mixture of linseed oil and safflower oil, and WNRS a mixture of linseed oil and safflower. Castor wax is present in the WNCB paint, and small amounts of triglycerides containing odd numbered fatty acids appear to be present in the WNRS paint. One previous study including the analysis of Winsor and Newton oil paint model paints indicated that water sensitivity (within the range of paints analysed) does not appear to relate to the type of oil, nor to the presence of castor wax, which does not appear to be consistently associated to water-sensitive paints.^14,42^ Castor wax has been previously identified in commercial paints, likely as a stabiliser or rheology modifier.^43,44^ Odd numbered fatty acids are widespread in fats from the animal kingdom, but are rare in plants,^45^ suggesting that small amounts of animal fats are present in WNRS. As for castor wax, animal fat might have been added to the paint formulation, but could also be a residue of the pigment preparation process.

**Fig. 4:**
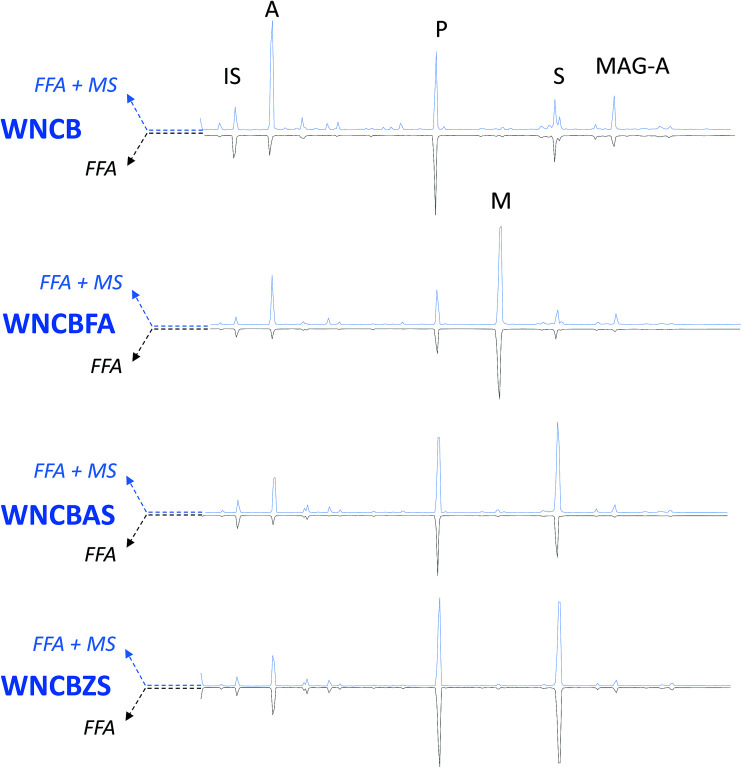
GC-MS chromatograms of WNCB model paints. IS: internal standard; A: azelaic acid; P: palmitic acid; M: margaric acid; S: stearic acid; MAG-A: azelaic acid monoacylglycerol. Blue traces – pointing up-chromatograms relative to free fatty and dicarboxylic acids + carboxylates of fatty and dicarboxylic acids (FFA + MS); black traces – pointing down – chromatograms relative to free fatty and dicarboxylic acids (FFA). All acids are separated in the form of silylesters.

**Fig. 5:**
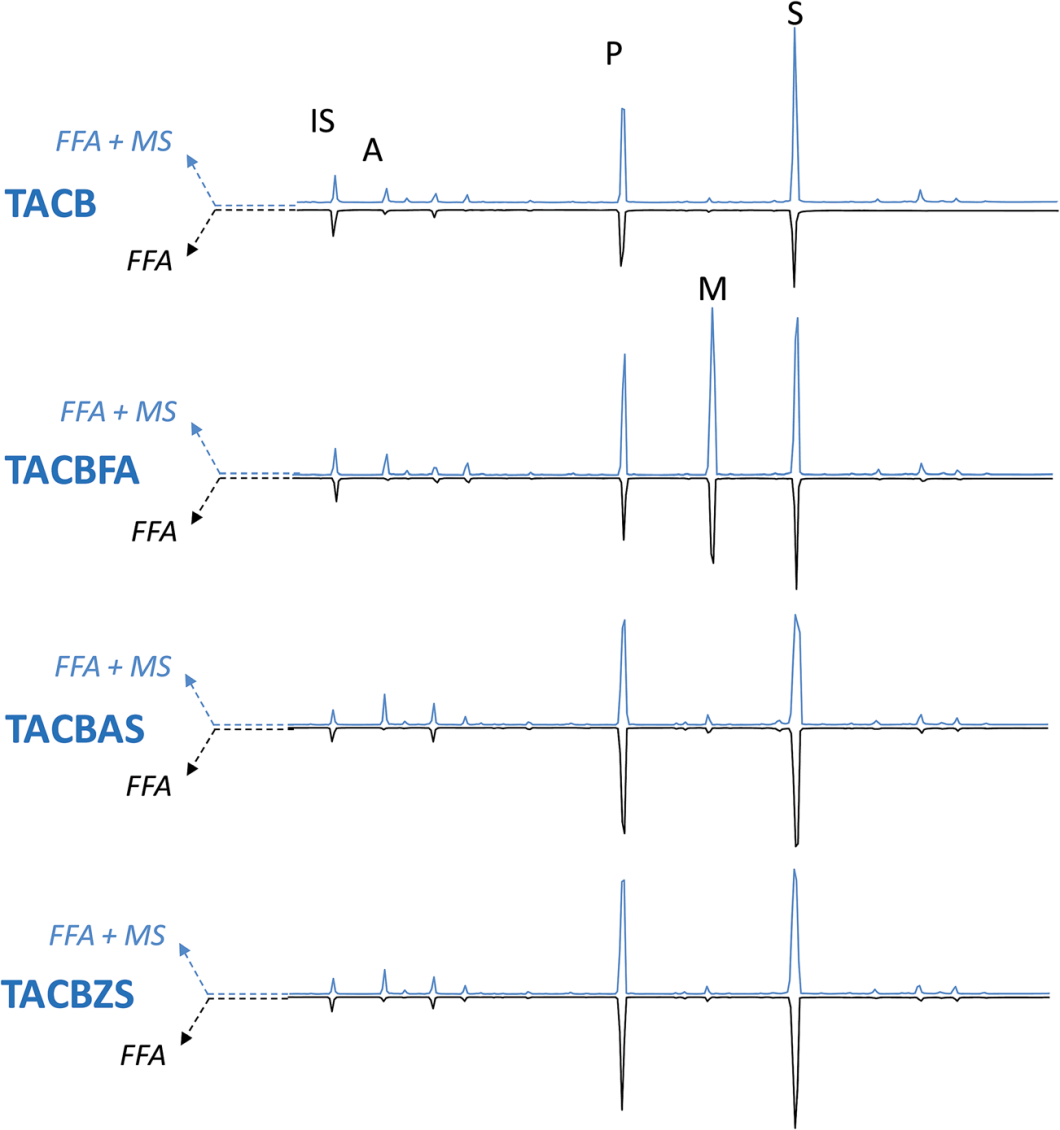
GC-MS chromatograms of TACB model paints. IS: internal standard; A: azelaic acid; P: palmitic acid; M: margaric acid; S: stearic acid. Blue traces – pointing up-chromatograms relative to free fatty and dicarboxylic acids + + carboxylates of fatty and dicarboxylic acids (FFA + MS); black traces – pointing down – chromatograms relative to free fatty and dicarboxylic acids (FFA). All acids are separated in the form of silylesters.

**Fig. 6:**
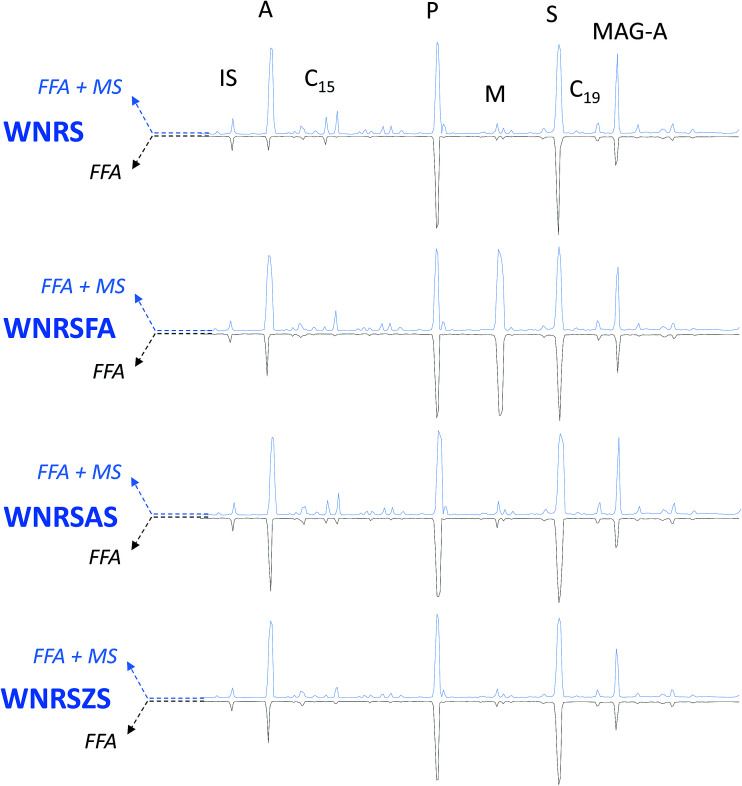
GC-MS chromatograms of WNRS model paints. IS: internal standard; A: azelaic acid; C_15_: pentadecanoic acid; P: palmitic acid; M: margaric acid; S: stearic acid; C_19_: nonadecanoic acid; MAG-A: azelaic acid monoacylglycerol. Blue traces – pointing up-chromatograms relative to free fatty and dicarboxylic acids + + carboxylates of fatty and dicarboxylic acids (FFA + MS); black traces – pointing down – chromatograms relative to free fatty and dicarboxylic acids (FFA). All acids are separated in the form of silylesters.

A newly developed GC-MS analytical procedure^22^ was adopted, to both qualitatively and quantitatively determine free fatty and dicarboxylic acids in the model paint samples, as well as free carboxylates of fatty and dicarboxylic acids (that are not bound to the polymeric network, nor to glycerides). This procedure entails two subsequent derivatisations on the same sample, first with HMDS, which is able to derivatise only free fatty and free dicarboxylic acids, and the second with BSTFA, which also derivatises the metal soaps of free fatty and dicarboxylic acids. Fig. 4–6 show the chromatograms of: (i) FFA – the fractions relative to the free fatty and dicarboxylic acids, and (ii) FFA + MS: the fraction relative to free fatty acids and dicarboxylic acids plus free carboxylates of fatty and dicarboxylic acids (MS).

**Fig. 7:**
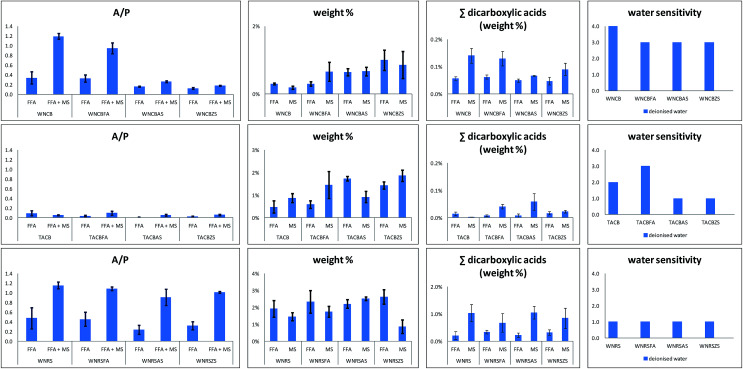
A/P: ratios between the relative content of azelaic acid and that of palmitic acid: weight%: FA – measure of the degree of hydrolysis, MS – measure of the degree of formation of metal soaps; ∑dicarboxylic acids (weight%): FFA relative content of free dicarboxylic acids. ∑dicarboxylic acids (weight%): MS – relative content of metal soaps of dicarboxylic acids; water sensitivity. Analyses were performed in triplicates and confidence intervals were calculated at a 95% confidence level.

Table 4 summarises the results of the quantitative analyses performed on the GC-MS data. Values reported are the average of measurements carried out on triplicate samples. Confidence intervals reported are those relative to a confidence level of 95%. Data reported in Table 4 were used to build the histograms reported in Fig. 7.

**Table 4:** Results of the quantitative analyses performed on the GC-MS data. A/P: ratio between the relative content of azelaic acid and palmitic acid; P/S: ratio between the relative content of palmitic acid and stearic acid; ∑dicarboxylic acids: sum of the relative content of dicarboxylic acids (azelaic, suberic and sebacic acids); weight%: measure of the degree of hydrolysis and degree of saponification – more details are reported in the text

Sample	Fraction	A/P	∑dicarboxylic acids (weight%)	P/S	weight%
WNCB	FFA	0.3 ± 0.1	0.06% ± 0.01%	1.5 ± 0.2	0.3% ± 0.0%
	FFA + MS	1.2 ± 0.1	0.14% ± 0.03%	2.4 ± 0.2	0.5% ± 0.1%
WNCBFA	FFA	0.3 ± 0.1	0.06% ± 0.01%	2.2 ± 0.3	0.3% ± 0.1%
	FFA + MS	0.9 ± 0.1	0.13% ± 0.03%	2.6 ± 0.7	0.8% ± 0.4%
WNCBAS	FFA	0.2 ± 0.0	0.05% ± 0.01%	0.7 ± 0.1	0.6% ± 0.1%
	FFA + MS	0.3 ± 0.0	0.07% ± 0.00%	0.7 ± 0.1	1.3% ± 0.4%
WNCBZS	FFA	0.1 ± 0.0	0.05% ± 0.01%	0.6 ± 0.0	1.0% ± 0.3%
	FFA + MS	0.2 ± 0.0	0.09% ± 0.02%	0.7 ± 0.0	1.9% ± 0.6%
TACB	FFA	0.1 ± 0.0	0.02% ± 0.01%	0.6 ± 0.0	0.5% ± 0.3%
	FFA + MS	0.1 ± 0.0	0.00% ± 0.00%	0.5 ± 0.0	1.4% ± 0.3%
TACBFA	FFA	0.0 ± 0.0	0.01% ± 0.00%	0.5 ± 0.0	0.6% ± 0.2%
	FFA + MS	0.1 ± 0.0	0.04% ± 0.01%	0.5 ± 0.0	2.0% ± 0.8%
TACBAS	FFA	0.0 ± 0.0	0.01% ± 0.01%	0.6 ± 0.0	1.7% ± 0.1%
	FFA + MS	0.1 ± 0.0	0.06% ± 0.03%	0.6 ± 0.1	2.7% ± 0.2%
TACBZS	FFA	0.0 ± 0.0	0.02% ± 0.01%	0.6 ± 0.0	1.4% ± 0.2%
	FFA + MS	0.1 ± 0.0	0.02% ± 0.00%	0.6 ± 0.1	2.8% ± 0.8%
WNRS	FFA	0.5 ± 0.2	0.21% ± 0.14%	0.8 ± 0.0	1.9% ± 0.5%
	FFA + MS	1.2 ± 0.1	1.03% ± 0.31%	0.8 ± 0.1	3.4% ± 0.5%
WNRSFA	FFA	0.5 ± 0.1	0.34% ± 0.06%	0.8 ± 0.0	2.3% ± 0.7%
	FFA + MS	1.1 ± 0.0	0.67% ± 0.34%	0.9 ± 0.1	4.1% ± 0.4%
WNRSAS	FFA	0.2 ± 0.1	0.23% ± 0.08%	0.7 ± 0.1	2.2% ± 0.2%
	FFA + MS	0.9 ± 0.2	1.05% ± 0.23%	0.7 ± 0.0	4.7% ± 0.3%
WNRSZS	FFA	0.3 ± 0.1	0.33% ± 0.10%	0.7 ± 0.0	2.6% ± 0.4%
	FFA + MS	1.0 ± 0.0	0.85% ± 0.37%	0.7 ± 0.1	6.0% ± 1.2%

The P/S values of the unmodified TACB and WNRS paints were noted as being quite low, particularly considering the type of oils used.^36^ This ratio, together with the presence of Zn (in TACB) and Al and Zn (in WNRS), strongly suggest that the paints contained Zn and Al stearates, which were likely to have been added by the paint manufacturers. Fig. 7 depicts the ratios between the relative content of azelaic acid and that of palmitic acid (A/P) obtained from the FFA and FFA + MS fractions.

Dicarboxylic acids are the final product of oxidation of drying oils, formed as a natural consequence of auto-oxidative reactions taking place during curing. Assuming that saturated monocarboxylic acids are stable over time,^46^ the A/P ratio classically calculated by GC/MS is related to the degree of oxidation of a paint.^20^ Free dicarboxylic and monocarboxylic acids in a paint are the result of the hydrolysis of triglycerides. Keeping this in mind, the A/P values in the FFA and FFA + MS fractions relate to the degree of hydrolysis of azelaic acid, and of the formation of its corresponding metal soap with respect to palmitic acid.^22^ In the TACB paint, the A/P ratios are never above 0.1. The WNRS paints were characterized by a high degree of formation of metal soaps containing azelaic acid with respect to palmitic acid – the A/P ratio values of the FFA + MS fractions were always significantly higher than those of the FFA fraction. In the WNCB paints, although the A/P ratio values of the FFA + MS fractions were always higher than those of the FFA fractions, the samples with added stearates contain significantly lower A/P values than the others.

In addition to the peaks ascribable to azelaic (A), palmitic (P), margaric (M) and stearic (S) acids, the chromatograms of the WNRS samples also show the presence of odd numbered fatty acids (pentadecanoic, margaric, and nonadecanoic acids – present in all chromatograms), confirming the observations of HPLC-MS analyses suggesting the presence of a fat of animal origin^45^ in this paint. The chromatograms of all W&N paints present a peak ascribable to 9-(2,3-dihydroxypropoxy)-9-oxononanoic acid (azelaic acid monoacylglycerol, MAG-A). The relative intensity of this peak is (in the majority of the samples) higher in the fraction derivatised with BSTFA than in the fraction derivatised with HMDS, suggesting that the free moiety of azelaic acid in the monoacylglycerol forms metal soaps. The presence of consistent amounts of metal soaps of azelaic acid monoacylglycerol leads to the assumption that azelaic acid, bound from one side to glycerol, is likely to be present as carboxylate also in diglycerides and triglycerides, which are not detected using gas chromatography due to their low volatility. This supports the recently presented model of an oil paint layer, according to which a significant fraction of carboxylate groups belong to the covalent part of the oil network, leading to the formation of a ionomer-like network.^28^

Fig. 7 also shows a measure of the degree of hydrolysis and the degree of formation of metal soaps of the model paint samples. The measure of the degree of hydrolysis of the model paint samples (FA weight% in Fig. 7) was calculated as the sum of the weight content of lauric (L), suberic (Sub), azelaic (A), myristic (M), sebacic (Seb), palmitic (P), oleic (O) and stearic (S) acids measured in the FFA fraction, normalised to the sample weight.

Measure of the degree of hydrolysis:
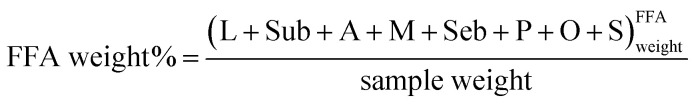


The measure of the degree of formation of soaps of fatty and dicarboxylic acids of the model paint samples (MS weight% in Fig. 7) was calculated as the difference between the sum of the weight content of lauric, suberic, azelaic, myristic, sebacic, palmitic, oleic and stearic acids measured in the FFA + MS fraction, and the sum of the weight content of lauric, suberic, azelaic, myristic, sebacic, palmitic, oleic and stearic acids measured in the FFA fraction, also normalised to the sample weight.

Measure of formation of soaps of fatty and dicarboxylic acids:
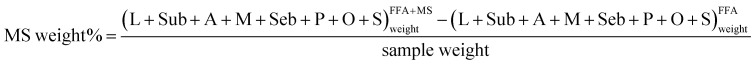


The determined amounts refer only to free fatty and dicarboxylic acids and their relative metal soaps, and thus not to those acids which are still bound to glycerides, nor to those that are incorporated into the polymeric/ionomer-like network. Also, the data refer to the sample weight – which accounts for the binder, pigment and any other additive present – and not to the organic content only, which is not known. As a result, the data from different paints cannot be compared quantitatively, although this can be done within the paints with the same pigment and within one brand.

The addition of free fatty acids (in this case margaric acid, which was not included in the calculations) causes an intrinsic increase of the free acidic moieties present in the paint, but does not appear to catalyse the hydrolysis of the paint (FFA weight% in Fig. 7 and Table 3 relative to samples WNCB/WNCBFA, TACB/TACBFA, and WNRS/WNRSFA). In samples WNCBFA and TACBFA, the added free fatty acids promptly formed metal soaps (see Fig. 4–6). Indicating the average value of the ratio between the amount of margaric acid in the FFA + MS fraction and that of the corresponding FFA fraction with MFFA + MS/MFFA, we obtained: MFFA + MS/MFFA(WNCB) = 2.1; MFFA + MS/MFFA(TACB) = 2.7; MFFA + MS/MFFA(WNRS) = 0.9 (Fig. 4–6 chromatograms relative to WNCBFA, TACBFA and WNRSFA).

Both Al and Zn stearates do appear to increase the relative content of free fatty acids in the paints, especially in the cobalt blue paints (see FFA weight% in Fig. 7 relative to samples WNCB/WBCBAS/WNCBZS, TACB/TACBAS/TACBZS, and WNRS/WNRSAS/WNRSZS). This could be due to the fact that technical stearates contain free fatty acids.^11,12^. Moreover it has been suggested that metal soaps might catalyse the hydrolysis of triglycerides.^47–49^

The relative content of free dicarboxylic acids (FFA∑ dicarboxylic acids (weight%) in Fig. 7) with respect to the sample weight was also calculated as the sum of the weight content of suberic, azelaic and sebacic acids measured in the FFA fraction, normalised to the sample weight.

Relative content of free dicarboxylic acids:
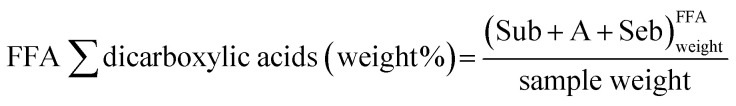


Differences in the content of free dicarboxylic acids n paints of the same series (same brand and some pigment) do not appear significant: the amount of dicarboxylic acids – which are partially water soluble does not appear to relate to water sensitivity, as the more water sensitive samples do not contain significantly higher amounts of free dicarboxylic acids than the less water sensitive samples. Previous research showed that irrespective of the overall degree of oxidation of the paint, ethanol extracted a relatively higher amount of free dicarboxylic acids from water-sensitive with respect to non-water sensitive paints.^19^ We can thus hypothesise that water sensitive samples are more accessible to water ingress, either by swelling the surface or capillary penetration, resulting in a more effective solubilisation of dicarboxylic acids. The condition of the paint may be explained by an insufficient degree of crosslinking of the dry film, making the paint layers less tightly bound, and thus susceptible to swelling by polar solvents. Conversely, we can hypothesise that non-water sensitive paints are characterised by a more extended polymeric network, and are thus not penetrated by water, resulting in a limited access of water to any free dicarboxylic acids present in the paint layers. As a result, the relatively high content of dicarboxylic acids in the extracts of water sensitive oil paint films may not be a direct cause of water sensitivity, but may be a consequence/symptom of the lack of formation of a well-developed polymeric/ionomeric paint system.

The relative content of metal soaps of dicarboxylic acids (MS ∑dicarboxylic acids (weight%) in Fig. 7) with respect to the sample weight was also calculated as the sum of the difference between the sum of the weight content of suberic, azelaic and sebacic acids measured in the FFA + MS fraction, and the sum of weight content of suberic, azelaic and sebacic acids measured in the FFA S fraction, normalised to the sample weight.

Relative content of metal soaps of dicarboxylic acids:
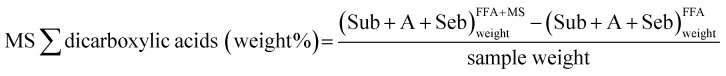


It was proposed that metal soaps of azelaic and other diacids form a relatively stable metal coordinated tri-dimensional network because of their chain building ability,^21^ contributing to paint stability. These data indicate that water sensitivity does not correlate with the relative amount of free metal soaps of dicarboxylic acids, as, within the same set of paints, the more water sensitive samples do not have lower proportions of free metal soaps of dicarboxylic acids.

An important observation noted from these data is that the relative content of free fatty acids and free metal soaps was below 10% by weight in all of the samples investigated. This amount is not sufficient to justify the intensity of the distinctive FTIR absorption band at 1711 cm^−1^ ascribable to free fatty acids (Fig. 2) in the WNRS sample, nor those of the metal soaps (absorption band at around 1590 cm^−1^ ascribable to metal carboxylates) in the samples WNCB and TACB (Fig. 1 and 2). To examine this further, fresh paints were prepared using haematite in linseed oil, and divided into two aliquots, stearic acid (10% w/w) was added to one aliquot and Zn stearate (10% w/w) was added to the other. Both were analysed by FTIR (IR spectra are reported in ESI); The IR spectra clearly show that the intensity of the C=O stretching vibration relative to free fatty acids (1710 cm^−1^) and that of the C=O stretching vibration relative to Zn stearate (1538 cm^−1^) is significantly lower than the intensity of the C=O stretching vibration relative to the oil glyceride (1743 cm^−1^)s. In addition, the IR absorptions ascribable to metal carboxylates in samples WNCB and TACB fall within the wavelength ranges assigned to amorphous metal carboxylates,^28,30^ which are not analysed by the GC-MS procedure, able only to detect carboxylates of fatty and dicarboxylic acids that are not bound to the polymeric/ionomeric network. The crosslinked network derives from the polymerization of polyunsaturated fatty acids. A polyunsaturated fatty acid, bearing more than one unsaturation, may crosslink at one carbon and oxidise at another carbon, leading to the formation of dicarboxylic acids, which are linked *via* a C–C or C–O–C bonds to other acids in the network. When combined, these observations lead us to conclude that the band at 1711 cm^−1^ in the WNRS sample, and the bands around 1590 cm^−1^ in the samples WNCB and TACB must be ascribed to acidic moieties and carboxylate groups, the majority of which are attached to the polymeric/ionomeric network, in which acid and metal coordinated carboxylate moieties coexist in a complex structure, which represents the main organic constituent of a mature paint film.

## 4. Conclusions

Free fatty and dicarboxylic acids, and carboxylates of fatty and dicarboxylic acids were determined qualitatively and quantitatively. The determined species represent the non-bonded fraction of a mature oil paint film. This information was interpreted in relation to the water sensitivity of the paints. Data clearly show that paints with water sensitivity rating from 1 to 4 (from non-water sensitive to highly water sensitive) are characterised by relatively similar amounts of free and dicarboxylic acids, and therefore no significant trend was observed for these parameters.

Comparison of spectroscopic data with the results of the GC-MS quantification of free fatty acids, free dicarboxylic acids, and their relative metal soaps indicated that most of the metal carboxylates and acidic moieties of free carboxylic acids in these paints are not part of the non-bonded fraction, but are associated with the polymeric/ionomeric network. In agreement with recent findings,^21,28,30,50^ we support the hypothesis of a model of polymeric/ionomeric network of a mature paint film composed of crosslinked and partially hydrolysed glycerides, a substantial portion of which are metal coordinated. The results of this study suggest that the nature of the polymeric/ionomeric network is a significant determining factor impacting on the development of water sensitivity. It has been previously shown that water sensitivity is dependent primarily on the pigment type.^14^ In this study we demonstrated that additives may also influence the molecular composition of a paint film (as observed from the analysis of molecular profiles of free carboxylic acids and their relative metal soaps), and may also affect water sensitivity: added Zn or Al stearates generally cause a small decrease of water sensitivity, while added free fatty acids do not show a consistent trend. Hence it is likely that not only pigments, but also additives can affect the curing process of the paint, influencing, together with external factors,^51^**F. Modugno, F. di Gianvincenzo, I. Degano, I. Bonaduce, K. J. van den Berg, “On the influence of relative humidity on the oxidation and hydrolysis of fresh and naturally aged oil paints “, 2018, in preparation. the degree of oxidation/crosslinking, and thus the nature of the mature polymeric/ionomeric network, which in turn contributes to the formation of water sensitive or water resistant paint films. Research is still necessary to investigate this complex system further, requiring the development of new analytical approaches, which can also investigate the molecular and physical composition of the polymeric/ionomeric network.

## Conflicts of interest

There are no conflicts to declare.

## Supplementary Material

RA-008-C7RA13364B-s001
