# Supplementary material for: A molecular study of modern oil paintings: investigating the role of dicarboxylic acids in the water sensitivity of modern oil paints
Source: RSC Adv. 2018 Feb 6;8(11):6001–12. doi: 10.1039/c7ra13364b (PMC9078243; doi:10.1039/c7ra13364b)
Supplement: RA-008-C7RA13364B-s001 [file RA-008-C7RA13364B-s001.pdf]

Electronic Supplementary Material (ESI) for RSC Advances.
This journal is © The Royal Society of Chemistry 2018

Electronic supplementary information

A molecular study of modern oil paintings: investigating the role of dicarboxylic acids in the water sensitivity of modern oil paints.

Donatella Banti[1], Jacopo La Nasa[2], Anna Lluveras Tenorio[2], Francesca Modugno[2], Klaas Jan van den Berg[3], Judith Lee[4], Bronwyn Ormsby[4], Aviva Burnstock[1], Ilaria Bonaduce[2*]

1. Courtauld Institute of Art, Somerset House, Strand, London, WC2R 0RN
2. Department of Chemistry and Industrial Chemistry, University of Pisa, via Moruzzi 13- 56124, Pisa
3. Cultural Heritage Agency of the Netherlands (RCE), Hobbemastraat 22, 1071 ZC, Amsterdam
4. Conservation Department, Tate, Millbank, London, SW1P 4RG

* corresponding author. email: ilaria.bonaduce@unipi.it

Triglyceride profiling of fresh paints by LC-MS.

Experimental

HPLC–ESI-Q-ToF analysis was carried out on samples of the fresh paints used for the model samples to investigate the composition of the binders. Samples (2-3 mg) were subjected to microwave-assisted extraction in a microwave oven Ethos One (Milestone, U.S.A., power 600 W), with 300 µL of a chloroform-hexane (3:2) mixture at 80°C for 25 min. The extracts were dried under a nitrogen stream, diluted with 600 µL of elution mixture, and filtered on a 0.45 µm PTFE filter (Grace Davison Discovery Sciences, U.S.A.) just prior to injection. HPLC-ESI-Q-ToF analyses were carried out using a 1200 Infinity HPLC, coupled with a Quadrupole-Time of Flight tandem mass spectrometer 6530 Infinity Q-ToF detector by a Jet Stream ESI interface (Agilent Technologies). The chromatographic column was a Poroshell 120 EC-C18 column (3.0 mm x 5.0 mm, 2.7 µm particle size) with a Zorbax eclipse plus C-18 guard column (4.6 mm x 12.5 mm, 5 µm particle size) at a flow rate of 0.3 $mL \cdot min^{-1}$ and at 45 °C. Aliquots of 10 µL were injected and the elution gradient was programmed using methanol/water 85:15 (eluent A) and iso-propanol (eluent B) as follows: 90 % A for 5 min, followed by a linear gradient to 90% B in 30 min (held for 10 min). Re-equilibration time for each analysis run was 10 min. ESI operating conditions: drying gas (N2, purity >98%): 350 °C and 10 $L \cdot min^{-1}$; capillary voltage 4.5 KV; nebulizer gas 35 psig; sheath gas (N2, purity >98%): 375 °C and 11 $L \cdot min^{-1}$. High resolution MS and MS/MS spectra were acquired in positive mode in the range 100-1700 m/z. The fragmentor was kept at 200 V, nozzle voltage 1000 V, skimmer 65 V, octapole RF 750 V. The MS/MS spectra presented in the text were obtained at 50 V. The collision gas was nitrogen (purity 99.999%). Data were collected by auto MS/MS acquisition with an MS scan rate of 1.03 spectra·sec-1 and an MS/MS scan rate of 1.05 spectra·sec-1; only one precursor was acquired per cycle (relative threshold 0.010%). The mass axis was calibrated daily using the Agilent tuning mix HP0321 diluted in water and acetonitrile (Agilent Technologies). MassHunter® Workstation Software (B.04.00) was used to carry out mass spectrometer control, data acquisition, and data analysis. The structures of the TAGs were identified by the evaluation of their exact mass, the interpretation of their tandem mass spectra and by comparison with previously published mass spectral data [1-3].

Discussion and results

HPLC-ESI-Q-ToF was used for triglyceride profiling [1] to identify the drying oil(s) used as paint binders. Figure 1 shows the HPLC-ESI-Q-ToF chromatograms of the fresh paint outs taken from the tubes used for the preparation of the model paint samples in 2006.

Triglycerides were named according to the following fatty acid abbreviations: C_{15}: pentadecanoyl ($C_{15:0}$); P: palmityl ($C_{16:0}$); Ln: linolenyl ($C_{18:3}$); L: linoleyl ($C_{18:2}$); O: oleyl ($C_{18:1}$); S: stearyl ($C_{18:0}$); Ar: arachidyl ($C_{20:0}$); B: behenyl ($C_{22:0}$); Li: lignoceryl ($C_{24:0}$).

In the chromatogram of TACB (Figure 1 a) LLL, LLP, LLO, LOP, OOL, OOP, and OOS together with the presence of triglycerides containing arachidic and lignoceric acids allowed the identification of safflower oil. WNCB (Figure 1b) contained linseed oil, highlighted by the presence of the TAGs LnLnLn, LnLnL, LnLnP, LnLL, LLnP, and POP. This sample also contained 2,3-di(12-hydroxy-octadecanoyloxy)propyl 12-hydroxy octadecanoate (12 hydroxy stearic acid triglyceride) which indicated the presence of castor wax [4]. Castor wax, which is used as a solid lubricant [4], a rheology modifier and/or a pigment dispersant, might have been added directly to the paint formulation, or might have derived from the pigment, as a residue from its preparation. Castor wax was also found in other W&N paints[5,6].

WNRS (Figure 1c), showed the presence of the same linseed oil markers highlighted in TACB. The chromatograms also show the presence of LLL, LLP, LLO, and LOP, pointing to the presence of safflower oil [2] (Figure 1 c), and PPC15, POC15, PSC15. Odd numbered fatty acids are reported to be widespread in fats from the animal kingdom, but are rare in plants [7]. This seems to point to the presence of small amounts of animal fat in WNRS. As for the case of castor wax in the W&N paint, it might have been added to the paint formulation, but could also be a residue of the pigment preparation process.

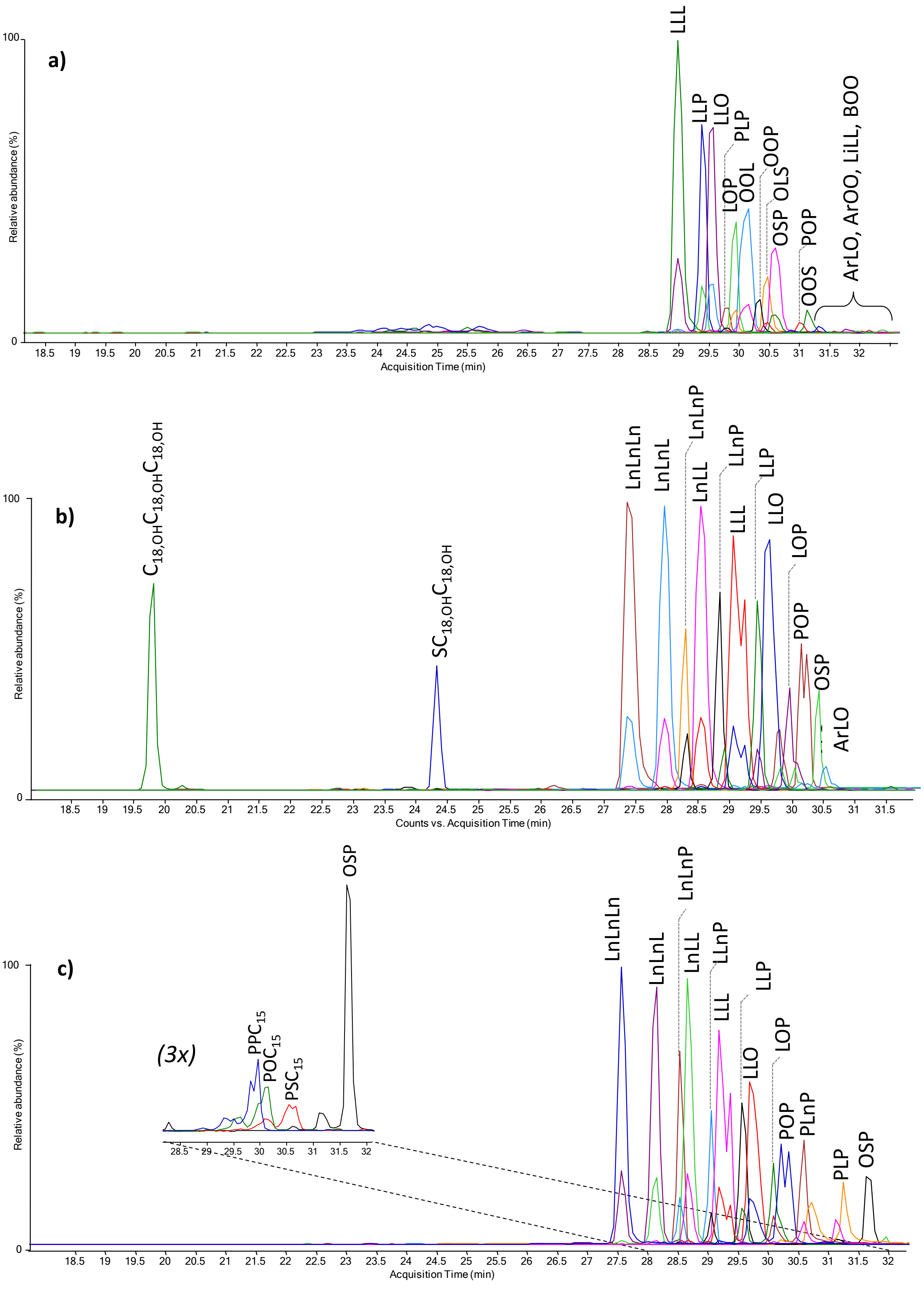

Figure 1. HPLC-ESI-Q-ToF extract ion chromatograms referring to the identified triglycerides in the paint tubes: a) TACB; b) WNCB; c) WNRS . C_{15}: pentadecanoyl (C15:0); P: palmityl (C16:0); Ln: linolenyl (C18:3); L: linoleyl (C18:2); O: oleyl (C18:1); S: stearyl (C18:0); Ar: arachidyl (C20:0); B: behenyl (C22:0); Li: lignoceryl (C24:0)

FTIR analysis of oil + pigment + additive mixtures.

Experimental

Fresh hematite paints were prepared in linseed oil, and divided into two aliquots. One aliquot was amended with added stearic acid (10% w/w) and the other with Zn stearate (10% w/w).

Discussion and results

The FTIR spectra of the two oil/pigment/additive mixtures are shown in Figure 2. The intensity of the C=O stretching vibration relative to free fatty acids (1710 cm^{-1})and that of the C=O stretching vibration relative to Zn stearate (1538 cm^{-1}) is significantly lower than the intensity of the C=O stretching vibration relative to the oil glycerides. The is not clearly distinguishable, and appears shown a broadening of the base of the peak of the the C=O stretching vibration relative to the oil glycerides.

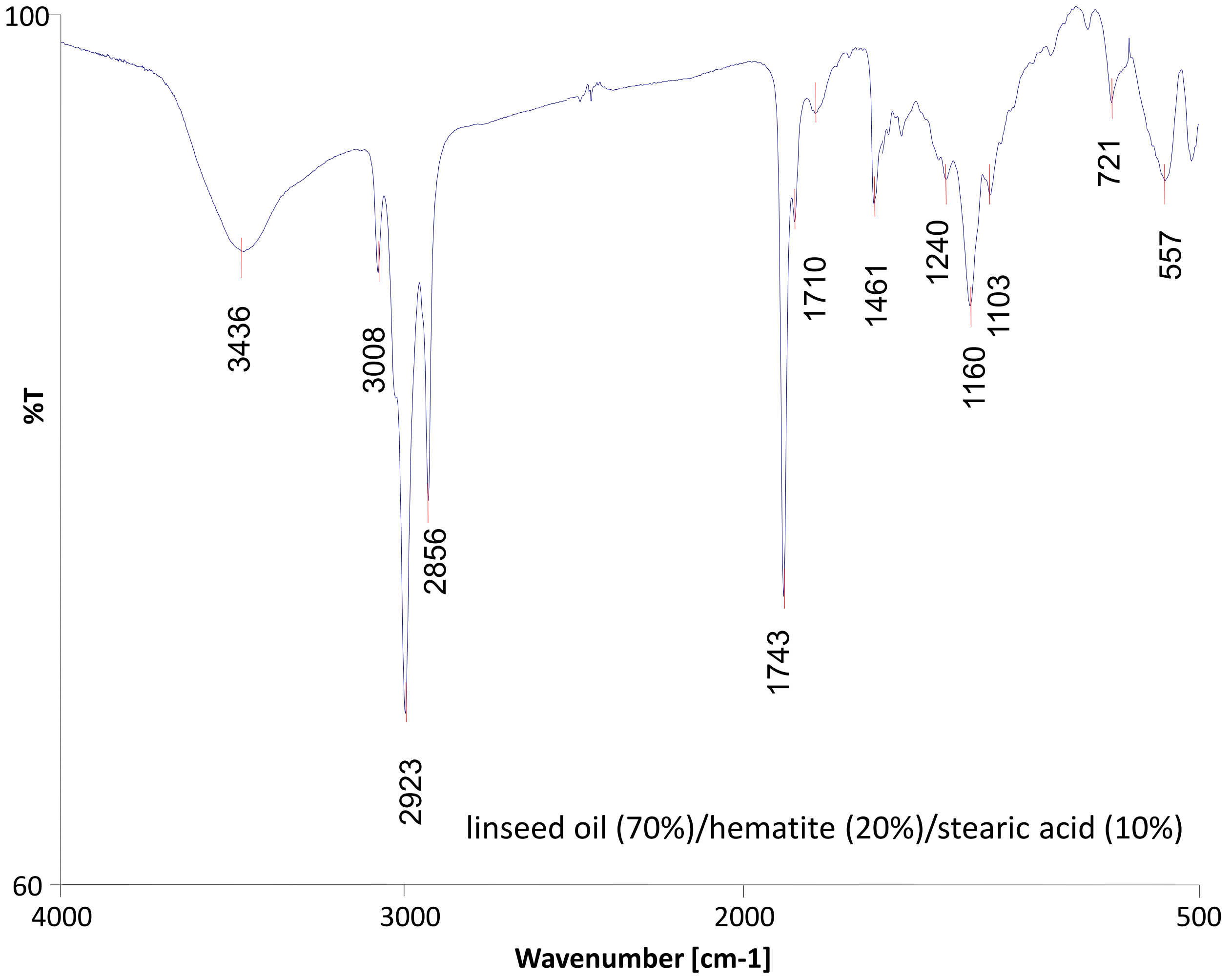

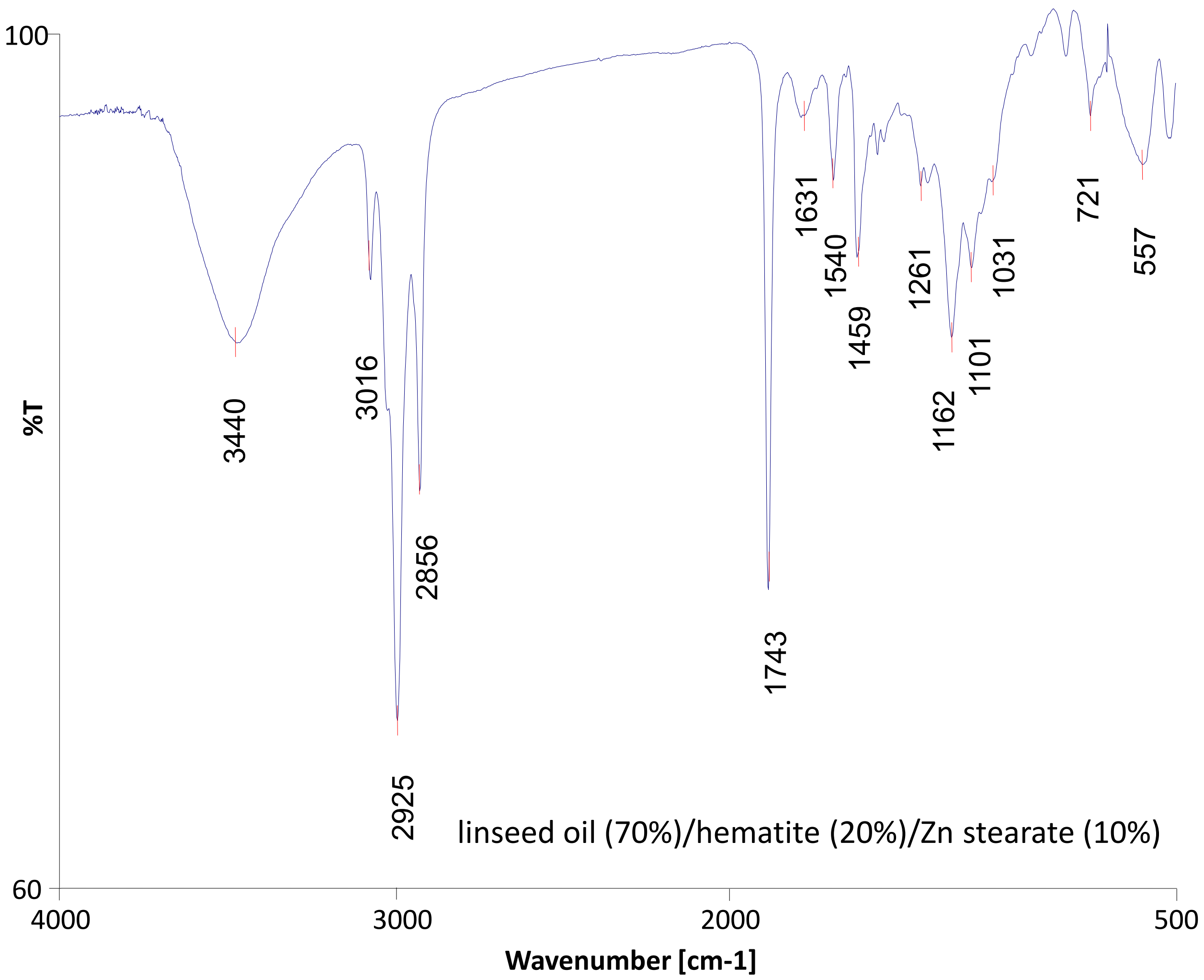

Figure 2. Top: FTIR spectrun of a hematite/linseed oil paint added with stearic acid (20%/70%/10% w/w); bottom: FTIR spectrum of a hematite/linseed oil paint added with Zn stearate acid (20%/70%/10% w/w).

Instrumentation

ATR-FTIR spectra were collected using a Perkin Elmer Spectrum 100 spectrometer. 16 scans were collected from 4000 to 500 cm^{-1} with a resolution of 4 cm^{-1}. Jasco Spectra Manager software was used for data analysis.

Acknowledgment

This work was performed within the context of the JPI CMOP project: “Cleaning Modern Oil Paints” (Heritage Plus Joint Call project 2015-2018). http://www.tate.org.uk/about-us/projects/cleaning-modern-oil-paints-0

Silvia Pizzimenti and Rita Carosi are kindly acknowledged for her help with FITR measurements.

References

(1) Degano, I.; La Nasa, J.; Ghelardi, E.; Modugno, F.; Colombini, M. P. *Talanta* **2016**, *161*, 62-70.
(2) La Nasa, J.; Ghelardi, E.; Degano, I.; Modugno, F.; Colombini, M. P. *Journal of Chromatography A* **2013**, *1308*, 114-124.
(3) Ilaria Degano; Nasa, J. L. **2016**, *374*, 1-28.
(4) Ogunniyi, D. S. *Bioresource Technology* **2006**, *97*, 1086-1091.
(5) Lee, J.; Bonaduce, I.; Modugno, F.; Ormsby, B.; van den Berg, K. J. *Microchemical Journal* **accepted for publication**.
(6) La Nasa, J.; Degano, I.; Modugno, F.; Colombini, M. P. *Analytica Chimica Acta* **2013**, *797*, 64-80.
(7) Diedrich, M.; Henschel, K. P. *Molecular Nutrition & Food Research* **1990**, *34*, 935-943.
